# Spongiosa Primary Development: A Biochemical Hypothesis by Turing Patterns Formations

**DOI:** 10.1155/2012/748302

**Published:** 2012-09-12

**Authors:** Oscar Rodrigo López-Vaca, Diego Alexander Garzón-Alvarado

**Affiliations:** ^1^Grupo de Investigación en Estudios y Aplicaciones de Ingeniería Mecánica (GEAMEC), Universidad Santo Tomás, Bogotá, Colombia; ^2^Grupo de Modelado y Métodos Numéricos en Ingeniería (GNUM), Departamento de Ingeniería Mecánica y Mecatrónica, Facultad de Ingeniería, Universidad Nacional de Colombia, Bogotá, Colombia

## Abstract

We propose a biochemical model describing the formation of primary spongiosa architecture through a bioregulatory model by metalloproteinase 13 (MMP13) and vascular endothelial growth factor (VEGF). It is assumed that MMP13 regulates cartilage degradation and the VEGF allows vascularization and advances in the ossification front through the presence of osteoblasts. The coupling of this set of molecules is represented by reaction-diffusion equations with parameters in the Turing space, creating a stable spatiotemporal pattern that leads to the formation of the trabeculae present in the spongy tissue. Experimental evidence has shown that the MMP13 regulates VEGF formation, and it is assumed that VEGF negatively regulates MMP13 formation. Thus, the patterns obtained by ossification may represent the primary spongiosa formation during endochondral ossification. Moreover, for the numerical solution, we used the finite element method with the Newton-Raphson method to approximate partial differential nonlinear equations. Ossification patterns obtained may represent the primary spongiosa formation during endochondral ossification.

## 1. Introduction

Most of the long bones of the mammals skeletal system are developed from a process called endochondral growth [1–4]. This process ends with the gradual production of bone from cartilage tissue during fetal development and postnatal growth. The process of ossification occurs from a hyaline cartilage tissue mold, which has a similar shape to the bone in a mature stage. The cartilage tissue molds are formed through the condensation of mesenchymal cells [5] followed by their differentiation into chondrocytes (cells that produce and maintain cartilage matrix) and secretion of typical components of the extracellular matrix of cartilage [6]. Once the mold of cartilage is formed, it is invaded initially in its center and then at each end by a mixture of cells that give rise to the appearance of primary and secondary centers of ossification, respectively, [7–9]. The ossification centers invade the cartilage gradually until it is completely replaced by bone tissue, except the articular surfaces. In this way, and eventually the bones reach their skeletal maturity [10]. The processes of endochondral development, growth, and elongation of the bones are made by the continuous addition of cartilage and subsequent replacement by bone tissue.

During the chondrocytes differentiation process, the matrix composition changes dramatically through the production of other components such as collagen type X, the expression of metalloproteinases, and subsequent calcification. At the same time the blood vessels invade the calcified cartilage bringing osteoblasts which build immature bone. Chondrocytes in the growth plate are subjected to the influence of excess extracellular factors including systemic and soluble local factors, as well as, extracellular matrix components. Several studies [9, 11–13] provide evidence that the proliferation of chondrocytes in the growth plate is under the control of a local closed loop that depends on the spatial and temporal location; and that mainly involves the molecular signals synthesized by chondrocytes: parathyroid hormone-related peptide (PTHrP), Indian hedgehog (Ihh), transforming growth factor (TGF*β*), bone morphogenetic proteins (BMPs), vascular endothelial growth factor (VEGF), matrix metalloproteinase type 9, known as gelatinase-B (MMP9), and the transcription factor RUNX2. They interact together in a feedback loop to regulate the rate at which chondrocytes leave the proliferative zone, differentiate hypertrophic cells and give way to immature bone formation [10, 11, 14]. The inappropriate balance in the expression of these molecules along with the ones that encode the collagens and other growth factors have been subject of studies as possible causes of impaired bone formation by the endochondral ossification mechanism [15–17].

The process of endochondral ossification has been studied for several years, and different models have been developed *in silico*, verified by histological reports, and *in vivo* experiments, which have tried to explain the process of bone formation through this mechanism [7–9, 13, 14, 18–21]. For example, Courtin et al. [18] in their work performed the comparison between the sequence of morphological events involved in embryonic bone formation and spatiotemporal characteristics of self-organization generated by a reaction-diffusion model related to the metabolism of the periosteal bone mineralization. In that article, 3D structures are obtained (by computer simulation) with a close resemblance to the primary internal architecture of the periosteum of long bones. The hypothesis of Courtin et al. is based on the role of self-organization of mineralization of bone metabolism, which gives rise to a well-organized space architecture. Subsequent research such as those by Garzón-Alvarado et al. [7–9, 22] have raised different hypotheses about the interaction of mechanical, cellular, and molecular factors that lead to the formation of secondary ossification centers in the epiphyses of long bones, and it also helped the development and bone growth and the primary bone formation. These hypotheses suggest that biological processes and interactions between different factors can be represented using mathematical models, where the chemical feedback among molecular reagent factors through reaction-diffusion mechanisms may explain the stable spatial patterns found in the origin of the secondary ossification centers and in the formation of cartilage canals. As far as the authors know, no research has been conducted on the action of different cellular, mechanical, and molecular factors on the development and production of primary spongiosa architecture during the endochondral ossification process, which is the basis for the production of trabecular bone. Similarly, there are no biochemical models involving reaction-diffusion systems with Turing instabilities and reaction equations based on the Schnakenberg model, that allow enlarge the knowledge and understanding of the development in the primary stage of the trabecular bone.

Therefore, this paper presents a hypothesis on the development of trabecular bone architecture. Starting from the assumption that the interaction of two molecular factors expressed by hypertrophic chondrocytes which through a reaction-diffusion mechanism generate a stable spatial-temporal pattern. This patterns lead to the formation of trabeculae present in the primary spongiosa tissue. This is the first model that attempts to explain the formation of primary spongiosa, which serves as the basis for defining a complete model of trabeculae formation at an early stage of skeletal development.

## 2. Molecular Mechanisms Present in the Endochondral Ossification

### 2.1. Molecular Factors

The sequential changes in the behavior of chondrocytes in the growth plate are highly regulated by systemic factors and the production of local factors. Growth hormone (GH) and thyroid hormone are systemic factors involved in endochondral ossification that regulate the behavior of the chondrocytes. GH is a peptide hormone-based protein that stimulates growth, cell reproduction and tissue regeneration. It is an important regulator of longitudinal bone growth [23]. The main effect of GH on chondrocytes, it is to stimulate their proliferation [24]. However, thyroid hormone is considered another systemic regulator of bone growth, and it stimulates production of collagen type II and X and alkaline phosphatase (ALP), which act as markers of bone mineralization.

On the other hand, local factors act as receptors to carry out intracellular signaling and selective activation of transcription factors of chondrocytes, such as insulin-like growth factors (IGF), which act as a local mediator of the effects of GH on cartilage growth. These factors are essential for embryonic skeletal development [25, 26], and in chondrocyte proliferation and/or hypertrophy. Parathyroid hormone-related peptide (PTHrP) is expressed by perichondrial cells at the initial stage of the chondrocytes proliferation. The PTHrP diffuses out of its place of production to act on cells carrying the receptor PTH/PTHrP [27]. The PTHrP keeps chondrocytes in a proliferative state and prevents hypertrophy [28]. The Indian Hedgehog (Ihh) is a local factor produced by the expression of prehypertrophic chondrocytes that stimulates the proliferation of chondrocytes and inhibits its hypertrophy. [14]. The Ihh stimulates osteoblastic differentiation of mesenchymal cells, which is essential for the formation of the periosteum surrounding the zone of hypertrophic chondrocytes. The formation of the periosteum precedes the formation of primary ossification center and it is maintained through its expansion [13, 29]. Bone morphogenetic proteins (BMPs) are another local factor. These are members of the transforming growth factor beta (TGF*β* superfamily), which is capable of inducing strongly immature bone formation, cartilage, and connective tissue [30]. Finally, within the local factors, there is the vascular endothelial growth factor (VEGF), which stimulates the process known as angiogenesis and acts as a vasodilator by increasing vascular permeability [31]. VEGF acts on vascular endothelial cells through specific tyrosine kinase membrane receptors, thereby regulating functions such as proliferation, differentiation, and migration of chondroblasts, osteoblasts, and osteoclasts [14]. During chondrocytes hypertrophy in the growth plate, VEGF is released and the extracellular matrix surrounding the hypertrophic cells, which begin the process of calcification. Then the extracellular matrix is invaded by blood vessels which provide nutrients and attract osteoblasts and osteoclasts that help the formation of the trabecular bone [32].

Transcription factors are mostly specific to a particular cell lineage and act as growth regulators of cell differentiation. They are predominantly expressed during skeletal development and its main function is to control cell proliferation or differentiation [33]. The transcription factor Runx2 is also called Cbfa1/Osf2/AML3/Til1/PPB2*α*A and is an essential protein in the differentiation of chondrocytes and osteblasts as well as the morphogenesis of the skeletal system [34]. The Runx2 controls bone mineralization of growing bones by stimulating osteoblast differentiation, promoting chondrocyte hypertrophy, and contributing to the migration of endothelial cells and vascular invasion. The Runx2 is expressed by chondrocytes in the early stages of hypertrophy and is maintained until terminal hypertrophic differentiation [35].

### 2.2. Regulation of Cartilage Matrix Degradation during Endochondral Ossification

The increase in cell volume experienced by the chondrocytes submitted to hypertrophy requires the degradation of the matrix that surrounds these cells. Moreover, the invasion of the ossification front requires an extensive (but selective) degradation of cartilage transverse columns surrounding the hypertrophic cells in the final state [10, 28]. There have been several studies to identify the proteolytic enzymes responsible of these events of matrix degradation and the cells responsible for its synthesis [36–38]. These studies have emphasized on the enzymes capable to degrade the two major protein components of the cartilage matrix, collagen type II and aggrecan. Within the growth plate is expressed selectively MMP13 by hypertrophic chondrocytes, which degrades collagen fibers and aggrecan [38, 39].

The MMP13 expression by chondrocytes is a prerequirement for the invasion of the growth plate by blood vessels, osteoclasts, and osteogenic cells. Therefore, these cells cannot enter in the empty gaps created by the death of hypertrophic chondrocytes until it degrades the septa of the cavities by the MMP13 [10]. Blood vessels invade the growth plate at the same time that the osteoblasts do it, which are necessary for the establishment of the primary ossification center. Thus, in the absence of MMP13 most of the cartilage matrix is not removed and there is no cell invasion in the bone marrow or bone matrix deposition in the remaining cartilage. The vascular invasion of the growth plate is facilitated by the vascular endothelial growth factor (VEGF), which is expressed by chondrocytes, and regulated with the hypertrophy under the control of Runx2 [10, 11]. On the other hand, the VEGF in the endochondral ossification, increases bone formation and decreases bone resorption [40, 41], indicating that VEGF regulates the production of MMP13.

## 3. Materials and Methods

### 3.1. Hypothesis Required for the Development of Primary Spongiosa Using Reaction-Diffusion Systems

The main hypothesis of this paper is based on the existence, within the endochondral ossification process, of the controlled interaction of two signaling molecules that diffuse and react chemically in the cartilage extracellular matrix, to carry out the formation of primary spongiosa from the growth plate. Accordingly, we assume the existence of a reaction-diffusion system where two primary molecules are involved, such as VEGF and MMP13, which can lead to a stable pattern in time and unstable in space, similar to the patterns present in the structure of the trabecular bone during endochondral ossification.

The presence of MMP13, which is released by hypertrophic chondrocytes, allows the degradation of cartilage matrix components (collagen and aggrecan) and leads to vascular invasion and ossification front [10, 36, 37]. This vascular invasion is facilitated by the presence of VEGF expressed by hypertrophic chondrocytes [31, 40, 42]. This means that when MMP13 and VEGF exist in all regions of the epiphyseal cartilage, having a high concentration of VEGF, there will be an adequate control of the invasion of endothelial cells, osteoclasts, chondroclasts, and osteoblasts, which are present in the primary ossification development [40]. Similarly, in those areas where there is a high concentration of MMP13, it will completely degrade the cartilage, giving rise to the trabecular bone architecture. Last statements are supported on Hiltunen et al. [43] studies. In their work it was injected a saline solution containing VEGF in the distal femur of white rabbits. Their results demonstrate that VEGF induces bone formation by increasing osteoblast activity and decreasing the resorption process. The resorption process is produced by both osteoclasts in bone and metalloproteinases (MMPs) in the growth plate. Therefore, it can be supposed that in the development of the architecture of the primary spongiosa, there must exist a regulation of MMP13 by the VEGF (inhibitory mechanism). So, it stops the degradation of the cartilage and begins the invasion front of ossification.

### 3.2. Model Description

**Figure 1:**
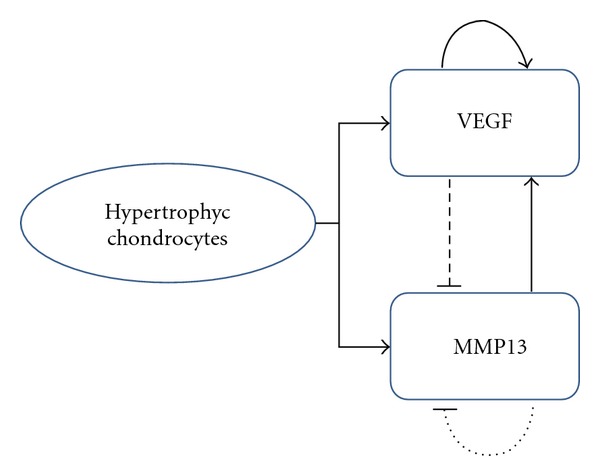
Control system of the molecular process. VEGF: vascular endothelial growth factor; MMP13: matrix metalloproteinase 13, the figure shows the ratio of the molecular signals produced by the hypertrophy of chondrocytes. The solid lines mean activation, dotted lines inhibition, continuous curved lines self-activation, and dotted curved lines self-inhibition.

The regulatory process proposed in this model is outlined in Figure 1, and it is based on an activator-substrate system (also called exhaustion model) (see Appendix A). The process indicates that there is a control loop between VEGF (activating factor) and the MMP13 (substrate), where the VEGF is self-activated and inhibits the production of MMP13 stopping the degradation process and giving a way to the mineralization of the remaining cartilage matrix [40]. On the other hand, we assume that the MMP13 is self-inhibited, but enables the production of VEGF, thereby this loop is called positive feedback system. The VEGF helps vascular invasion and bring with it the osteogenic cells that allow the construction of the primary spongiosa. The MMP13 allows the degradation of the cartilage matrix and the subsequent invasion of the cartilage by the ossification front.

The regulatory mechanism is modeled by reaction-diffusion equations. The reaction term (synthesis of soluble extracellular factors) is considered dependent on the concentration of the reactants and the presence of hypertrophic chondrocytes. According to this, the hypothesis is based on the origin of the patterns presented in the primary spongiosa. It could correspond, from a mathematical point of view, to the patterns that occur in the Turing space when two chemical reactants interact.

The definition of the relationships shown in Figure 1 can be quantified by means of equations which provide local changes of the extracellular soluble factors and the production rate of bone:

(1a) ∂SVEGF∂t  =  CCH(α1−μSVEGF+γ0SVEGF2SMMP13)+DVEGF∇2SVEGF,

(1b) ∂SMMP13∂t  =  CCH(α2−γ0SVEGF2SMMP13)+DMMP13∇2SMMP13,

(1c) ∂cBone∂t  =  ηSVEGFnSVEGFn+Sumbraln·  TarTar+tr,

where *C*_CH_ is the concentration of hypertrophic chondrocytes, *S*_VEGF_, *S*_MMP13_ represent the concentrations of VEGF and MMP13, respectively. The remaining terms are model parameters. *α*_1_ and *α*_2_ are terms that quantify the production of each molecular factor by hypertrophic chondrocytes, *μ* is a constant that quantifies inhibition of the VEGF production by it excess, *γ*_0_ regulates the nonlinear interaction between the concentration of MMP13-VEGF quantifying the concentration or molecular inhibition of each molecular factor, and *D*_VEGF_ and *D*_MMP13_ are the diffusion coefficients of VEGF and MMP13, respectively. In the biological interpretation of the above equations the term *γ*_0_ *S*_VEGF_^2^ *S*_MMP13_ represents the nonlinear activation of *S*_VEGF_ (production of VEGF by the presence of MMP13) and the nonlinear consumption of *S*_MMP13_ (by the presence of VEGF). Equation (1c) represents the activation of bone production rate by the presence of high amounts of VEGF, which is regulated as time goes on. In this equation *c*_Bone_ indicates the production of bone per unit of volume due to the concentration and distribution of VEGF within the domain. *η* is a constant that regulates the production of bone over time, *S*_Umbral_^*n*^ represents the value of the concentration of VEGF with which begins the process of ossification, *T*_*a*_ is the time required for the process of cartilage calcification, and *t* ^*r*^ represents the time that limits the production of bone.

### 3.3. Solution of the Reaction-Diffusion Equations System Using the Finite Element Method

To solve the set of ((1a), (1b), and (1c)), we used the finite element method, with tetrahedral elements. Due to the nonlinearity of the terms included in the model, we used the Newton-Raphson method to solve the problem of time evolution of the concentration of VEGF (*S*_VEGF_) and MMP13 (*S*_MMP13_). The time integration was performed using the trapezoidal rule.

#### 3.3.1. Weak Formulation

Let ((1a), (1b), and (1c)) be rewritten as

(2) ∂SVEGF∂t−CCH(α1−μSVEGF+γ0SVEGF2SMMP13) −DVEGF∇2SVEGF=0,∂SMMP13∂t−CCH(α2−γ0SVEGF2SMMP13) −DMMP13∇2SMMP13=  0,∂cBone∂t−ηSVEGFnSVEGFn+Sumbraln·TarTar+tr=0.

Using weighted residuals, the system of (2) takes the form

(3) ∫Ωw1(∂SVEGF∂t−CCH(α1−μSVEGF+γ0SVEGF2SMMP13)      −DVEGF∇2SVEGF)dΩ=0,∫Ωw2(∂SMMP13∂t  −CCH(α2−γ0SVEGF2SMMP13)   −DMMP13∇2SMMP13)dΩ=0,∫Ωw3(∂cBone∂t−ηSVEGFnSVEGFn+Sumbraln·  TarTar+tr)dΩ=0,

where *Ω* represents the domain of the problem which is limited by the boundary Γ. *w*_1_, *w*_2_, and *w*_3_ are weight functions. Using Green's theorem and weakening the system of (3), the residue of the problem is given by

(4) rSVEGF=∫Ωw1∂SVEGF∂tdΩ−∫Ωw1CCHα1dΩ+∫Ωw1CCHμSVEGFdΩ−∫Ωw1CCHγ0SVEGF2SMMP13dΩ+∫ΩDVEGF∇w1∇SVEGFdΩ−∫Γw1DVEGF(∇SVEGF·n)dΓ=0,rSMMP13=∫Ωw2∂SMMP13∂tdΩ−∫Ωw2CCHα2dΩ+∫Ωw2CCHγ0SVEGF2SMMP13dΩ+∫ΩDMMP13∇w2∇SMMP13dΩ−∫Γw2DMMP13(∇SMMP13·n)dΓ=0,rCBone=∫Ωw3(dcBonedt     −ηSVEGFnSVEGFn+Sumbraln·TarTar+tr)dΩ=0.

Defining null flow conditions on the contour of the problem, the border-related terms in (4) are canceled, so the residue is expressed as

(5) rSVEGF=∫Ωw1∂SVEGF∂tdΩ−∫Ωw1CCHα1dΩ+∫Ωw1CCHμSVEGFdΩ−∫Ωw1CCHγ0SVEGF2SMMP13dΩ+∫ΩDVEGF∇w1∇SVEGFdΩ=0,rSMMP13=∫Ωw2∂SMMP13∂tdΩ−  ∫Ωw2CCHα2dΩ+∫Ωw2CCHγ0SVEGF2SMMP13dΩ+∫ΩDMMP13∇w2∇SMMP13dΩ=0,rCBone=∫Ωw3(∂cBone∂t−ηSVEGFnSVEGFn+Sumbraln·TarTar+tr)  dΩ=0.

To discretize the finite element solution we use approaching functions written as a linear combination of orthogonal functions as shown in the following:

(6) $$\begin{array}{c} S_{\text{VEGF}}^{e}=N_{v}S_{\text{VEGF}},\\ S_{\text{MMP13}}^{e}=N_{m}S_{\text{MMP13}},\\ c_{\text{Bone}}^{e}=N_{c}c_{\text{Bone}}, \end{array}$$

where **N**_*v*_, **N**_*m*_, and **N**_*c*_ represent the shape functions which depend only on the space used for the formulation, **S**_VEGF_ and **S**_MMP13_ are the values of *S*_VEGF_ and *S*_MMP13_ in the nodal points, and the superscript *e* indicates the discretization of the finite element variable. For the weighting functions we used the Bubnov-Galerkin formulation, indicating that the functions *w* take the same form of approximation functions **N**.

Substituting (6) in (5), we obtain the residual vector in its discrete form (7), where $\underline{r_{\text{VEGF}}^{e}}$, $\underline{r_{\text{MMP13}}^{e}}$ and $\underline{r_{C_{\text{Bone}}}^{e}}$ are residue vectors for each equation and ∇**N** is the gradient vector of the approximation functions

(7) rSVEGFe_=∫ΩNT∂SVEGFe∂tdΩ−∫ΩNTCCHα1dΩ+∫ΩNTCCHμSVEGFedΩ−∫ΩNTCCHγ0(SVEGFe)2SMMP13edΩ      +∫ΩDVEGF∇NT∇SVEGFedΩ,rSMMP13e_=∫ΩNT∂SMMP13e∂tdΩ−∫ΩNTCCHα2dΩ+NT+∫Ωw2CCHγ0(SVEGFe)2SMMP13edΩ+∫ΩDMMP13∇NT∇SMMP13edΩ,rCBonee_=∫ΩNT(∂cBone∂t    −ηSVEGFnSVEGFn+Sumbraln·  TarTar+tr)dΩ.

Using the time discretization equation based on the Crank-Nicolson equations, the equations in (7) are transformed into the following:

(8) rSVEGFe_=∫ΩNTS~VEGFe−SVEGFeΔtdΩ+α(∫ΩDVEGF∇NT∇S~VEGFedΩ−∫ΩNTCCHα1dΩ+∫ΩNTCCHμS~VEGFedΩ−∫ΩNTCCHγ0(S~VEGFe)2S~MMP13edΩ      )t+Δt +(1−α)(∫ΩDVEGF∇NT∇SVEGFedΩ−∫ΩNTCCHα1dΩ+∫ΩNTCCHμSVEGFedΩ−∫ΩNTCCHγ0(SVEGFe)2SMMP13edΩ      )t,rSMMP13e_=∫ΩNTS~MMP13e−SMMP13eΔtdΩ+α(∫ΩDMMP13∇NT∇S~MMP13edΩ−∫ΩNTCCHα2dΩ+NT+∫Ωw2CCHγ0(S~VEGFe)2S~MMP13edΩ)t+Δt +(1−α)(∫ΩDMMP13∇NT∇SMMP13edΩ−∫ΩNTCCHα2dΩ+NT+∫Ωw2CCHγ0(SVEGFe)2SMMP13edΩ)t,rCBonee_=∫ΩNTc~Bone−cBoneΔtdΩ−(∫ΩNTηSVEGFnSVEGFn+Sumbraln·TarTar+trdΩ)t+Δt −(∫ΩNTηSVEGFnSVEGFn+Sumbraln·TarTar+trdΩ)t,

where ${\tilde{S}}_{\text{VEGF}}^{e}$ and ${\tilde{S}}_{\text{MMP13}}^{e}$ are the nodal values *S*_VEGF_^*e*^ and *S*_MMP13_^*e*^ evaluated over time *t* + Δ*t*. *α* is a parameter characteristic of the integration method.

Using (8) is possible to determine each of the terms of the tangent stiffness matrix, as shown in the following:

(9) ∂rSVEGFe_∂S~VEGFe=1Δt∫ΩNTNdΩ+α(∫ΩDVEGF∇NT∇NdΩ  +∫ΩCCHμNTNdΩ−  ∫ΩNTNCCHγ02(S~VEGFe)S~MMP13edΩ      ),∂rSVEGFe_∂S~MMP13e=−α∫ΩNTNCCHγ0(S~VEGFe)2dΩ,∂rSMMP13e_∂S~VEGFe=α∫ΩNTNCCHγ02(S~VEGFe)S~MMP13edΩ,∂rSMMP13e_∂S~MMP13e=1Δt∫ΩNTNdΩ+α(∫ΩDMMP13∇NT∇NdΩ    +∫ΩNTNCCHγ0(S~VEGFe)2dΩ),∂rCBonee_  ∂c~Bone=1Δt∫ΩNTNdΩ.

The nodal values *S*_VEGF_^*e*^ and *S*_MMP13_^*e*^ in time *t* + Δ*t* can be approximated by the iterative algorithm of Newton-Raphson, as described in (10). In this equation, Δ*S*_VEGF_^*e*^ and Δ*S*_MMP13_^*e*^ represent the difference of the nodal values in two consecutive iterations

(10) $$\begin{array}{c} \left[\begin{array}{cc} \begin{array}{c} \frac{\partial \underline{r_{S_{\text{VEGF}}}^{e}}}{\partial {\tilde{S}}_{\text{VEGF}}^{e}} \end{array} & \begin{array}{c} \frac{\partial \underline{r_{S_{\text{VEGF}}}^{e}}}{\partial {\tilde{S}}_{\text{MMP13}}^{e}} \end{array}\\ \frac{\partial \underline{r_{S_{\text{MMP13}}}^{e}}}{\partial {\tilde{S}}_{\text{VEGF}}^{e}} & \frac{\partial \underline{r_{S_{\text{MMP13}}}^{e}}}{\partial {\tilde{S}}_{\text{MMP13}}^{e}} \end{array}\right]\left[\begin{array}{c} \Delta S_{{}_{\text{VEGF}}}^{e}\\ \Delta S_{\text{MMP13}}^{e} \end{array}\right]=-\left[\begin{array}{c} \begin{array}{c} \underline{r_{\text{VEGF}}^{e}} \end{array}\\ \underline{r_{\text{MMP13}}^{e}} \end{array}\right]. \end{array}$$

### 3.4. Numerical Implementation

The set of ((1a), (1b), and (1c)) were implemented and solved numerically using the finite element method with a Newton-Raphson scheme. The two examples given were solved in a laptop of 4096 MB and 800 MHz processor speed. Computer simulation was carried out in an incremental iterative scheme that allows solving, computationally, the evolution of both the concentration of molecular factors (*S*_VEGF_, *S*_MMP13_) and the production of immature bone. Initially the growth plate is assumed as a structural matrix with an initial concentration of chondrocytes in a hypertrophic stage 65.000 cell/mm^3^. The initial concentrations of VEGF and MMP13 are distributed randomly in the growth plate, with a disturbance of 10% on the steady-state, the concentration is given by (*S*_VEGF_, *S*_MMP13_) = (1.0,0.9) [ng/mL] [43] (see Appendix A). The selection of random initial conditions around the steady state is similar to the event of molecular expression of the hypertrophic chondrocytes in an area of ossification. The flow condition for each molecular factor in the boundary is assumed to be null, this is because these conditions are assumed periodic over the domain. The parameter values used are shown in Tables 2(a) and 2(b), the justification of all the parameters used in the illustrated examples is given in Appendix B.

## 4. Results and Discussion

To verify the potential of the proposed model in predicting the primary spongiosa architecture, two numerical tests were conducted in a three-dimensional cubic element with a length of 0,22 mm. This is an average length of a minimal element of the trabecular bone in postmineralized fetal stage, as presented by Ruimerman et al. [44] in their work of modeling and adaptation of trabecular bone. The parameters of the reaction-diffusion model were selected in order to obtain structures with a periodicity in accordance with the one present in the trabecular bone [28, 44, 45]. In the finite element mesh we employed 17.756 nodes and 16.625 tetrahedral elements. In all the simulations we used incremental steps of Δ*t* = 0.1 therefore this means that the simulation time is measured in seconds and every step Δ*t* in the biological process corresponds to 7 seconds.

**Figure 2:**
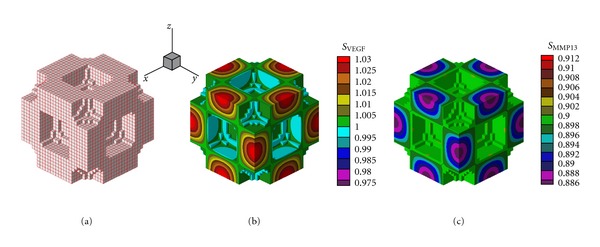
(a) Architecture of the primary spongiosa obtained by the proposed R-D model with a wave number (2,2,2). (b) Spatial distribution of VEGF concentration at the end of the ossification process. (c) Spatial distribution of MMP13 concentration at the end of the ossification process.

**Figure 3:**
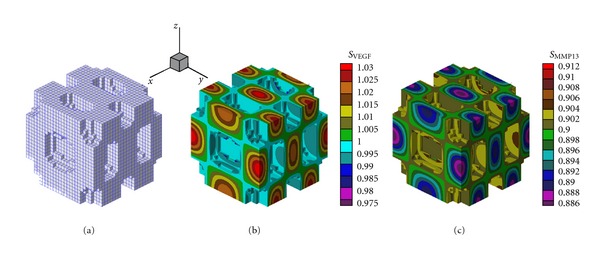
(a) Architecture of the primary spongiosa obtained by the proposed R-D model with a wave number (4,2,2). (b) Spatial distribution of VEGF concentration at the end of the ossification process. (c) Spatial distribution of MMP13 concentration at the end of the ossification process.

As a result of chemical interaction between the two molecular factors (reactants) and by the numerical results it was determined spatial patterns stable over time. The concentration of molecular factors in the cartilage and the action of the diffusive processes allows the formation of a pattern that is replicated throughout the domain. The architecture of the primary spongiosa obtained by the proposed RD model depends on the parameters used in ((1a), (1b), and (1c)) (see Tables 2(a) and 2(b)), so you can get structures with wave number (2,2,2) as shown in Figure 2 and with wave number (4,2,2) in Figure 3  (see Appendix B). The wave numbers allow either to define or to condition the frequency and distribution of the number of pores in a specific direction [9, 22, 46]. The appearance of either microstructure of the primary spongiosa, depends on the location of the parameters in the reaction diffusion equation ((1a), (1b), and (1c)) in the space of Turing. The location of certain points in this space, determine spatial patterns as shown in the results of this article.

The results in Figure 2 show the formation of two half-waves in each of the *x*, *y*, and *z*, directions, while Figure 3 shows the formation of four half-waves in the *x* direction and 2 half-waves in the *y* and *z* directions, respectively. Figures 2(b), 2(c), 3(b), and 3(c) show the results for the organization of VEGF and MMP13 after the stabilization of the reaction-diffusion process, note that in the areas of greatest concentration of VEGF is produced cartilage calcification and in areas of greatest presence of MMP13 degradation occurs (empty space).

**Figure 4:**
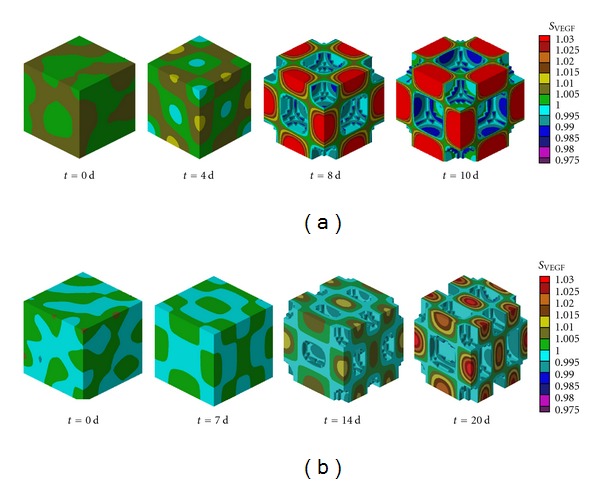
Temporal evolution of the concentration of VEGF (*S*_VEGF_) by the reaction-diffusion mechanism. (a) Wave mode (2,2,2). (b) Wave mode (4,2,2).

From the reaction-diffusion mechanism, it can be determined the change in the concentration of VEGF and MMP13 for each time step, as shown in Figure 4. The concentrations of VEGF (*S*_VEGF_) and MMP13 (*S*_MMP13_) within the cartilage evolve according to its diffusivity, its interaction, and its expression by the hypertrophic chondrocytes. So, the VEGF and the MMP13 are concentrated in high amounts in specific areas of the growth plate, allowing the formation of regular patterns similar to what happened in different biological models [9, 47–51].

**Figure 5:**
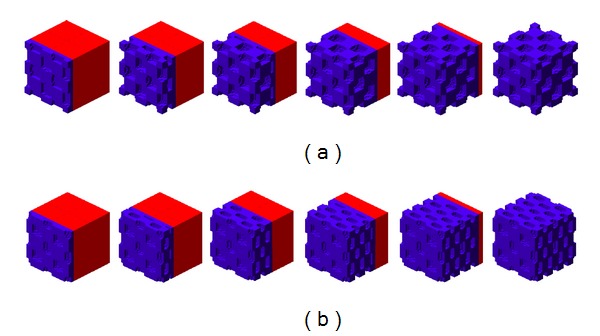
Primary spongiosa architecture produced by the interaction of VEGF and MMP13 by means of a reaction-diffusion mechanism. (a) Architecture generated by the use of a wave mode (2,2,2). (b) Architecture generated by the use of a wave mode (4,2,2). The blue areas represent ossified cartilage, and the red areas represent unossified cartilage.

The architecture of the primary spongiosa in a cubic element of length 0.44 mm is shown in Figure 5. In this figure we can observe the regular patterns for two different wave modes (2,2,2) and (4,2,2). Likewise, it details the advance in the front of ossification in different time steps, allowing the invasion of the cartilage by osteogenic cells that produce degradation and calcification. This will promote the formation of primary trabeculae, which subsequently undergo the bone remodeling processes, product of the stress distribution on bone tissue.

The development of primary spongiosa has been the subject of study in recent years, but there is no clarity about the biological and mechanical factors affecting its formation. It was found that the structure of the trabecular tissue from its beginning (fetal age) to adulthood has different patterns in its formation, as well as, a variation in the trabecular density product of the bone remodeling process and the effect of mechanical loads. The proposed model assumes that the formation of patterns is due to the interaction through a reaction-diffusion system of two molecules (VEGF, MMP13) during endochondral ossification. The results presented show that the patterns self-organize along the domain used, as shown in Figures 2, 3, and 5. These structures represent the architecture of the primary spongiosa considering only biochemical effects. The results obtained in this work can be compared to the structure used by Ruimerman et al. [44], which, show self-organized repetitive patterns that serve as the basis for the maintenance and adaptation of mature trabecular structure.

The production of molecular factors that act as activator-substrate by the differentiation of prehypertrophic chondrocyte, are not necessarily the only factors expressed by these chondrogenic cells, which probably affect considerably the process of ossification, even the chondrocytes are not the only cells that act in this process. However, the proposed model only focuses on the formation of primary spongiosa architecture and not the entire calcification process, in which bone cells also operate as osteoclasts and osteoblasts. In the complete calcification process the model should not only incorporate chemical influences (bioregulatory model) but also involves loads and restrictions at the boundary (mechanical effects) as well as additional biochemical factors that must be taken into account. For example, Ruimerman et al. [44], Jang and Kim [52], Renders et al. [53], and Coelho et al. [54] have proved in their works that mechanical factors play an important role in the development, adaptation, and maintenance of the trabecular bone structure. Moreover, from the viewpoint of the biochemical factors, works such as those of Brouwers et al.[14] have evaluated the potential of three growth factors, PTHrP, Ihh, and VEGF, that interact and regulate the tissue differentiation and development of a long bone. 

Much has been learned in recent years about the cellular and molecular mechanisms that guide the different events which allow the production of immature bone through endochondral ossification mechanism [6, 10, 11, 14, 19, 27, 31, 33, 45, 55–58]. Nevertheless, there are still concerns about the relationship and interaction of different events to allow ossification and endochondral growth.

In this paper we presented a bioregulatory model based on a set of reaction-diffusion equations to predict the formation of primary spongiosa architecture. The application of the reaction-diffusion model with parameters in the Turing space is an area of constant work and controversy in biology. Garzón-Alvarado et al. [8, 9, 22, 46], Courtin et al. [18] and Cramping and Maini [47], used in their researches reaction-diffusion models to simulate different biological processes, finding in their results, that the use of these systems may explain many complex biological phenomena where pattern formation is a constant variable.

## 5. Conclusions

In this paper we presented the development of a biochemical model involving reaction-diffusion systems with instabilities in the Turing space. This model attempts to explain the generation of primary spongiosa in the process of endochondral ossification, an event that is not yet fully understood, due to the amount of biological, mechanical and biochemical effects that are present. The model involves the controlled interaction of two important molecular factors, such as VEGF and MMP13, present in the development and bone formation.

The work presented in this paper illustrates and supports the validity of the reaction-diffusion models to describe the processes occurring during a complex event of pattern formation in bone biology. From the results presented, it can be concluded that the chemical feedback between the two reactants molecular factors (activator-substrate) could be an explanation from a set of possible factors that determine the complex spatial patterns found in the origin of the architecture of the primary spongiosa. However, it is clear that these results have been obtained with a mathematical model based on assumptions and simplifications that should be discussed.

The hypothesis presented suggests that the origin of the primary spongiosa is internally controlled by cartilage cells. This is achieved through two biochemical reagents, VEGF and MMP13. These are not the only factors acting in the endochondral ossification, there are many others, among which count Ihh, PTHrP, Runx2, BMP, [10, 11, 14, 31] and likely influence similarly to the trabecular bone formation. Until now there has been a great effort to fully understand the role of each of these substances, how they interact and what processes they regulate. It is possible that VEGF-MMP13 are not the factors that control the entire process of endochondral ossification, but the existence of an activator-substrate mechanism ensures high stability for the development of this biological process. By the other side, the hypothesis raised is based entirely on the cycle of bone formation through the endochondral ossification mechanism, as presented in [6, 10, 28]. Afterwards, in the bioregulatory model (see Figure 1) it is taken the MMP13 as an agent responsible for the degradation of cartilage septa and as a promoter of the activation of VEGF, which fosters vascularization of the cartilage and subsequent calcification. However, we do not discard the possibility of a new bioregulatory model where it considers the counter case presented. This indicates that the activation of MMP13 may be produced by the action of VEGF, suggesting that cartilage degradation initially requires vascular invasion, as presented in the work of Pufe et al. [59].

In the development of the model it is assumed for the initial conditions that the activating factor is released by hypertrophic chondrocytes, as well as the substrate; however, the type of spatial instability obtained is independent of the initial conditions. Nevertheless, this model is very stable and robust with respect to the initial conditions and the range of parameters.

Finally, despite all the limitations and simplifications, the proposed mathematical model is able to reproduce in detail the architecture of the primary spongiosa, allowing variation in porosity and thickness of the trabeculae. The proposed model will serve as the basis for the formation of the secondary spongiosa architecture, from the bone remodeling process, observing the action of bone cells and the different mechanical effects that determine the orientation of the trabeculae.

## Figures and Tables

**Table 1:** Vibration modes for the nondimensional model of Schakenberg with *a* = 0.1, *b* = 0.9, *T* = 1, and *L* = 0.22 mm.

*d*	*γ*	*a*	*b*	Wave mode
8,6123	346,3578	0,1	0,9	(2,2,2)
8.5736	700,4675	0,1	0,9	(4,2,2)

**Table:** (a)

Parameter	Value	Units
μ	0,6562	mm^3^/cell day
γ_0_	6,56 (10^11^)	mm^9^/cell day g^2^
α_1_	6,56 (10^−8^)	g/cell day
α_2_	5,91 (10^−6^)	g/cell day
*D*_MMP13_	5,9 (10^−4^)	mm^2^/s
*T*	11,7	Minutes

**Table:** (b)

Parameter	Value	Units
μ	1,327	mm^3^/cell day
γ_0_	1,327 (10^12^)	mm^9^/cell day g^2^
α_1_	1,327 (10^−7^)	g/cell day
α_2_	1,19 (10^−6^)	g/cell day
*D*_MMP13_	5,9 (10^−4^)	mm^2^/s
*T*	11,7	Minutes

## Appendices

### A. Reaction-Diffusion Equations

Turing [60] proved, theoretically, that a chemical system of reaction diffusion could spontaneously, evolve in a heterogeneous spatial pattern from a uniform initial state in response to infinitesimal perturbations [51, 59]. His model is a system of two special partial differential equations, which are known as the two components of a reaction-diffusion system. In the most general case where several components are present the system has the form:

(A.1) $$\begin{array}{c} \frac{\partial u_{i}}{\partial t}=D_{i}\nabla^{2}u_{i}+f_{i}\left(u_{1},u_{2},\ldots ,u_{m}\right),\;x\;\in \;\Omega ,t\geq 0, \end{array}$$

where the unknown functions *u*_*i*_(*x*, *t*) can be interpreted as a reagent of concentration, the term *D*_*i*_∇^2^ *u*_*i*_ describes the diffusion reagent and *f*_*i*_ is a smooth function (usually polynomial or rational in *u*_*i*_) that describes a non-linear chemical interaction between the reagents. Ω refers to the bounded domain and in the system operates some initial boundary conditions. Turing also introduced some key concepts such as an activator and an inhibitor, so it was assumed that cellular states are discrete and can be modified by special chemical reagents.

It is known that for two components of a reaction-diffusion system, the Turing instability, which leads to the formation of patterns [60], it is possible to find it for two types of systems characterized by the signs of the stability matrix (Jacobian) in a positive homogeneous stationary system. The signs of the coefficients of the linearized dynamic system around a fixed point, namely the defined curves by the functions *f*(*u*, *v*) = 0 and *g*(*u*, *v*) = 0 in the plane *u* − *v*, for a system of two chemical components, are cut in the point (*u*_0_, *v*_0_), contain relevant information on the mechanisms of destabilization of the homogeneous solution. It can be shown that when the signs of the stability matrix are given by

(A.2) $$\begin{array}{c} J\;\equiv \;\left(\begin{array}{cc} f_{u} & f_{v}\\ g_{u} & g_{v} \end{array}\right)\;=\;\underset{\text{Tipo I}}{\underset{︸}{\left(\begin{array}{cc} + & +\\ - & - \end{array}\right)}}\;\;o\;\underset{\text{Tipo II}}{\underset{︸}{\;\left(\begin{array}{cc} + & -\\ + & - \end{array}\right)}}. \end{array}$$

It is possible to find a Turing instability if the reaction rates and diffusion coefficients allow the fixed point (*u*_0_, *v*_0_) to be stable to small homogeneous perturbations, and be able to become unstable to inhomogeneous perturbations, these conditions can be summarized mathematically by four inequalities:

(A.3) $$\begin{array}{c} f_{u}+g_{v}<0,\\ f_{u}\;g_{v}-f_{v}g_{u}>0,\\ d\;f_{u}+g_{v}>0,\\ {\left(d\;f_{u}+g_{v}\right)}^{2}\geq 4d\left(f_{u}g_{v}-f_{v}g_{u}\right), \end{array}$$

where *d* represents the nondimensional coefficient of the diffusivities *d* = *D*_*v*_/*D*_*u*_. These inequalities are based on the linear stability analysis, that is, from the study of the eigenvalues (*λ*) of the linearized dynamics.

The stability matrix indicates what type of Turing instability can be found, for the case presented in this paper, the matrix corresponds to type 1 which identifies a reaction-diffusion system activator-substrate.

According to (A.1), for a reaction-diffusion system of two components, we have

(A.4) $$\begin{array}{c} \frac{\partial u}{\partial t}=D_{1}\nabla^{2}u+f\left(u,v\right),\\ \frac{\partial v}{\partial t}=D_{2}\nabla^{2}v+g\left(u,v\right), \end{array}$$

where we have the following terms of reaction:

(A.5) $$\begin{array}{c} f\left(u,v\right)=a-u+u^{2}v,\;\;g\left(u,v\right)=b-u^{2}v. \end{array}$$

For the point of equilibrium, we have

(A.6) $$\begin{array}{c} f\left(u,v\right)=0,\\ g\left(u,v\right)=0. \end{array}$$

The Jacobian (stabilization matrix) is given by

(A.7) $$\begin{array}{c} J=\left[\begin{array}{cc} \frac{\partial f}{\partial u} & \frac{\partial f}{\partial v}\\ \frac{\partial g}{\partial u} & \frac{\partial g}{\partial v} \end{array}\right]. \end{array}$$

Therefore,

(A.8) $$\begin{array}{c} \frac{\partial f}{\partial u}=2uv-1,\\ \frac{\partial f}{\partial v}=u^{2},\\ \frac{\partial g}{\partial u}=-2uv,\\ \frac{\partial g}{\partial v}=-u^{2}. \end{array}$$

For a steady point (*u*_0_, *v*_0_), we have *f*(*u*, *v*) = 0 and *g*(*u*, *v*) = 0; therefore, *u* and *v* are given by

(A.9) $$\begin{array}{c} u=a+b,\\ v=\frac{b}{{\left(a+b\right)}^{2}}. \end{array}$$

Replacing (A.9) into (A.8), we have

(A.10) $$\begin{array}{c} \frac{\partial f}{\partial u}=\frac{2b}{a+b}-1,\\ \frac{\partial f}{\partial v}={\left(a+b\right)}^{2},\\ \frac{\partial g}{\partial u}=\frac{-2b}{a+b},\\ \frac{\partial g}{\partial v}=-{\left(a+b\right)}^{2}. \end{array}$$

Taking *a* = 0.1 and *b* = 0.9 [9] and replacing in (A.10), we have

(A.11) $$\begin{array}{c} \frac{\partial f}{\partial u}=0.8,\\ \frac{\partial f}{\partial v}=1,\\ \frac{\partial g}{\partial u}=-1.8,\\ \frac{\partial g}{\partial v}=-1. \end{array}$$

Therefore, the stability matrix takes the following structure of signs:

(A.12) $$\begin{array}{c} J\equiv \left(\begin{array}{cc} f_{u} & f_{v}\\ g_{u} & g_{v} \end{array}\right)=\left(\begin{array}{cc} + & +\\ - & - \end{array}\right). \end{array}$$

Indicating that the substance *u* is self-activated and inhibits the substance *v*, while, the substance *v* is self-inhibited and activates the substance *u*, forming an activator-substrate system.

### B. Estimation of the Values for the Parameters

The set of ((1a), (1b), and (1c)) corresponds to a coupled system, where the equations that correspond to the molecular factors (1a) and (1b) are extended reaction-diffusion equations, similar to a Turing system, which has a controlled diffusion due to instabilities. For (*D*_VEGF_, *D*_Ihh_ ≠ 0), the controlled diffusion by instabilities appear for some combination of parameters [9, 47, 61], this defines a domain in the parameters space called Turing space. To get a Turing space is necessary a linear stability analysis of the reaction-diffusion system on the homogeneous solution, which is obtained by forcing (∂*S*_VEGF_/∂*t*)(*D*_VEGF_ = 0) = 0 and (∂*S*_MMP13_/∂*t*)(*D*_MMP13_ = 0) = 0, and leads to (*S*_VEGF_*, *S*_MMP13_*) = ((*α*_1_ + *α*_2_)/*μ*  , *α*_2_ *μ* ^2^/*γ*_0_(*α*_1_+*α*_2_)^2^). The linear analysis allows you to find the spatial patterns of the linearized solution and the range of parameters that ensure the emergence of such specific patterns [61]. Therefore, the solution can be expressed as (*S*_VEGF_, *S*_MMP13_) = (*u* + *S*_VEGF_*, *v* + *S*_MMP13_*), where *u* and *v* are small perturbations in each molecular factor, respectively. From (1a) and (1b) the results of the linear analysis allow to write the following inequalities:

(B.1) CCH(2γ0SVEGF∗SMMP13∗−γ0SVEGF∗−μ)<0,CCH2(γ0SVEGF∗2(μ−2γ0SVEGF∗SMMP13∗)   +2γ02SVEGF∗3SMMP13∗)>  0,CCH(DMMP13(2γ0SVEGF∗SMMP13∗−μ) −DVEGFγ0SVEGF∗2)>  0,CCH2(DMMP13(2γ0SVEGF∗SMMP13∗−μ)−DVEGFγ0SVEGF∗2)2 ⋯−4DVEGFDMMP13CCH2(γ0SVEGF∗2               ×(μ−2γ0SVEGF∗SMMP13∗)               +2γ02SVEGF∗3SMMP13∗)>0.

These inequalities define a domain in the parameter space, known as the Turing space, where the uniform steady state (*S*_VEGF_*, *S*_MMP13_*) is linearly unstable.

If we express (1a) and (1b) in a nondimensional form (Schnakenberg equation) and as a function of small perturbations of the molecular factors (*S*_VEGF_, *S*_MMP13_), respectively, through (*u*, *v*), we can obtain

(B.2) $$\begin{array}{c} \frac{\partial u}{\partial t}=\gamma \left(a-u+u^{2}v\right)+\nabla^{2}u,\\ \frac{\partial v}{\partial t}=\gamma \left(b-u^{2}v\right)+d\nabla^{2}v, \end{array}$$

where the parameters can be identified for the two models and their relationship:

(B.3) $$\begin{array}{c} a=\frac{\alpha_{1}}{\mu }\sqrt{\frac{\gamma_{0}}{\mu }},\\ b=\frac{\alpha_{2}}{\mu }\sqrt{\frac{\gamma_{0}}{\mu }},\\ d=\frac{D_{\text{MMP13}}}{D_{\text{VEGF}}},\\ T=\frac{L^{2}}{D_{\text{VEGF}}},\\ \gamma =\frac{L^{2}}{D_{\text{VEGF}}}\mu C_{\text{CH}},\\ {\text{S}}_{\text{VEGF}}={\text{S}}_{\text{MMP13}}=\sqrt{\frac{\mu }{\gamma_{0}}}, \end{array}$$

where *T* is the characteristic time of the biological process and *L* is the characteristic length of the dimensional model. Therefore, defining (*γ*, *d*, *a*, and *b*) it is possible to obtain the eigenvalues and eigenvectors of the set of (B.3) and from them, the different spatial patterns corresponding to different wave numbers.

In the case of the proposed dimensional model it is necessary to define the nondimensional parameters (*L*, *D*_VEGF_, *D*_MMP13_, *C*_CH_, *μ*, *γ*, *α*_1_, and *α*_2_). To estimate these values, we consider for this work some experimental evidence:

- The typical concentration of VEGF in human tissue is *S*_VEGF_ = *S*_MMP13_ = 1 ng/mL [43].
- The study domain is a cubic three-dimensional element of side *L* = 0,22 mm [44].
- Concentration of hypertrophic chondrocytes in the proximal femoral epiphysis is *C*_CH_ = 65000 cells/mm^3^ [8].
- The coefficient of diffusivity for VEGF is 6,9 × 10^−5^ mm^2^/s [14].

To reproduce the patterns present in the primary trabecular bone architecture with the proposed model, it is necessary that all parameters are in the Turing space and that they satisfy the restrictions (A.3). Therefore, taking the values for *S*_VEGF_, *L*, and *D*_VEGF_, and the set of values for *γ*, *d*, *a*, and *b* [8, 22] (see Table 1) that satisfy the restrictions of Turing, and using the relations of (24), we get the set of values to be used for the solution of ((1a), (1b), and (1c)) (see Tables 2(a) and 2(b)).
